# ETS-NOCV decomposition of the reaction force for double-proton transfer in formamide-derived systems

**DOI:** 10.1007/s00894-017-3564-9

**Published:** 2017-12-22

**Authors:** Piotr Talaga, Mateusz Z. Brela, Artur Michalak

**Affiliations:** Department of Theoretical Chemistry, Faculty of Chemistry, Jagiellonian University, Gronostajowa 2, 30-387 Krakow, Poland

**Keywords:** Reaction force decomposition scheme, Natural orbitals of chemical valence, ETS-NOCV, Double proton transfer, Formamide, Thioformamide

## Abstract

The analysis of the electronic-structure changes along IRC paths for double-proton-transfer reactions in the formamide dimer (R1), formamide–thioformamide system (R2), and the thioformamide dimer (R3) was performed based on the extended-transition-state natural orbitals for chemical valence (ETS-NOCV) partitioning of the reaction force, considering the intra-fragments strain and the inter-fragments interaction terms, and further—the electrostatic, Pauli-repulsion and orbital interaction components, with the latter being decomposed into the NOCV components. Two methods of the system partitioning into the fragments were considered (‘reactant perspective’/bond-formation, ‘product perspective’ / bond-breaking). In agreement with previous studies, the results indicate that the major changes in the electronic structure occur in the transition state region; the bond-breaking processes are, however, initiated already in the reactant region, prior to entering the TS region. The electrostatic contributions were identified as the main factor responsible for the increase in the activation barrier in the order R1 < R2 < R3.

**Electronic supplementary material:**

The online version of this article (10.1007/s00894-017-3564-9) contains supplementary material, which is available to authorized users.

## Introduction

The reaction paths on the Born–Oppenheimer potential-energy surface, and the corresponding potential-energy profiles of chemical reactions, *E(ξ*), where *ξ* represents the reaction coordinate, are among the vital concepts of chemistry. The derivatives of the energy with respect to the reaction coordinate define the *reaction force* [1] (negative energy gradient) and the *reaction force constant* [2] (second energy derivative, i.e., negative gradient of the reaction force). The usefulness of these concepts in analysis of the reaction mechanisms has been demonstrated in numerous examples originating from the research groups of Politzer, Toro-Labbé, and others [1–11]. In particular, the reaction force provides a basis for the definition of distinctive regions of the reaction pathway: the reactant (R), transition state (TS), and product (P) region; usually, most of the electronic changes takes place in the TS region, while structural changes dominate in the R and P regions [5–7]. The reaction force constant provides information on the active mode driving the reaction as well as on the synchronicity of the changes in the electronic structure due to bond-breaking and bond-formation [2, 10].

In chemistry, it is natural to refer to the fragments of the reactive system when analyzing the chemical reaction, e.g., reactants, products, atoms, functional groups, etc. Partitioning of the system into chemical fragments in combination with energy decomposition schemes can provide more detailed insight into the mechanisms of the chemical reactions or the factors controlling the activation barriers. The energy decomposition schemes can be quite straightforwardly used to decompose the reaction force as well, by differentiating the corresponding energy terms with respect to the reaction coordinate. Recently, Politzer et al. [11] discussed the forces driving and retarding chemical reactions based on the decomposition of the reaction force utilizing the energy-partitioning within the Activation Strain Model (ASM) proposed by Bickelhaupt [12–15]. In this approach, the terms associated with the structural deformation of the reactants (strain, deformation) and the interaction between the chemical fragments are considered. In a recent article [16], we proposed to further decompose the interaction component of the reaction force, based on the Ziegler–Rauk energy decomposition scheme (*extended transition state*, ETS; *energy decomposition analysis*, EDA) [17–19] and the *extended-transition-state natural orbitals for chemical valence* (ETS-NOCV) approach [20]; the ETS-NOCV decomposition of the reaction force was used in analysis of the water assisted HCN/CNH isomerization [16], and in the metal-assisted intra-molecular proton transfer in thymine [21].

**Fig. 1:**
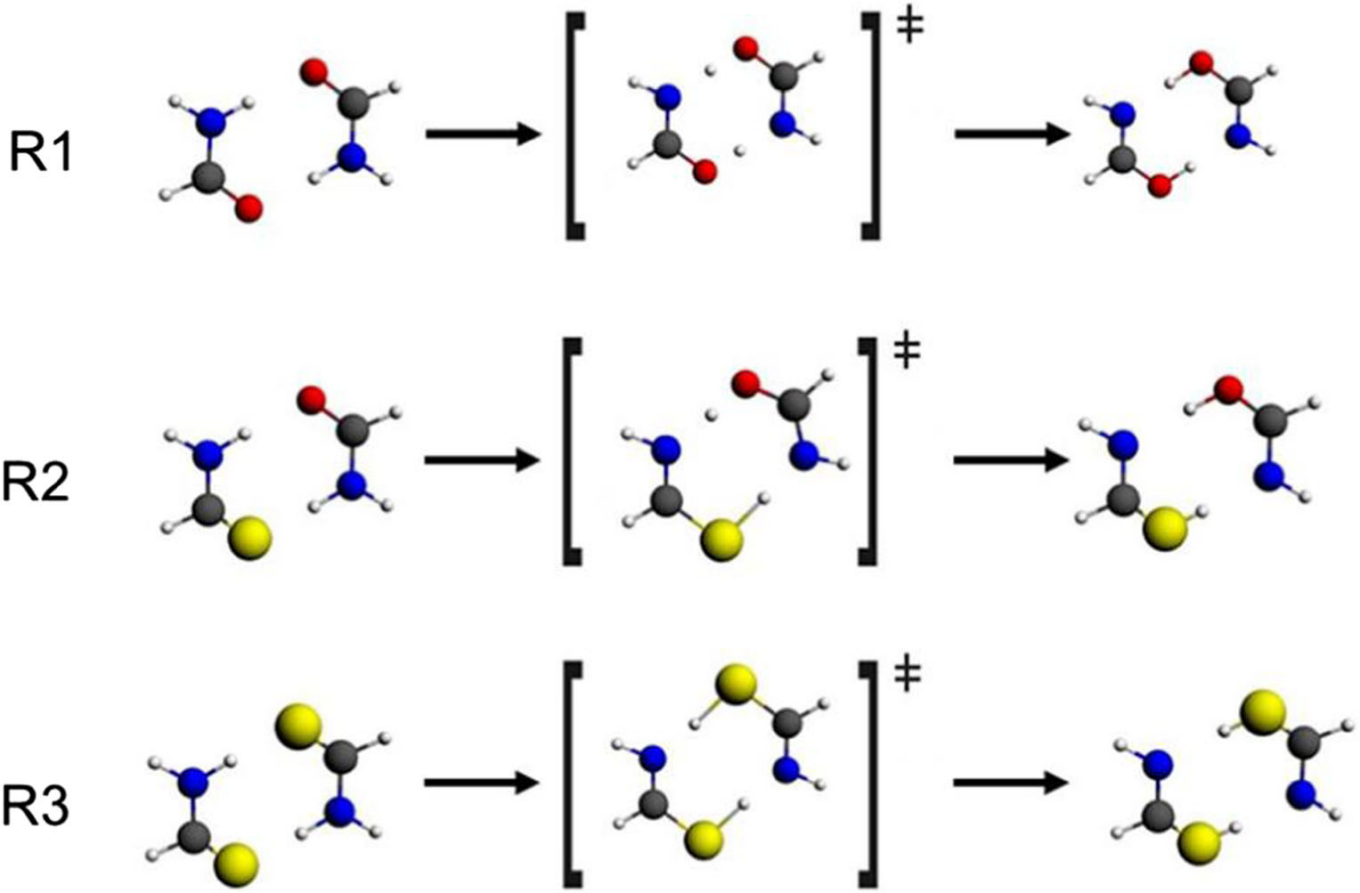
The double proton-transfer reactions in formamide dimer (R1), formamide–thioformamide system (R2), and thioformamide dimer (R3). The structures of reactants, transition states, and products are shown

The main goal of the present work is to analyze the ETS-NOCV components of reaction force for the reaction pathways of the double proton transfer in formamide dimer (R1), formamide–thioformamide system (R2), and thioformamide system (R3) (see Fig. 1). The double-proton transfer in formamide dimer was a subject of numerous theoretical studies; in particular, results of analysis of the reaction force and the reaction electronic flux were recently presented by Hargis et al. for R1 [22], as well as by Inostroza-Riviera et al. for R1-R3 [23]. Our objective is to analyze the driving and retarding components of the reaction force emerging from ETS-NOCV decomposition, as well as to identify the factors responsible for increase of the activation barrier in the order: R1 < R2 < R3.

## Theoretical background

The reaction force [1–11] is defined as the negative derivative of the potential energy *E(ξ*) with respect to the reaction coordinate *ξ*:

1 $$ F\left(\xi \right)=-\frac{d E\left(\xi \right)}{d\xi} $$

For the potential-energy-surface points representing the reactant(s), transition state, and product(s), the reaction force vanishes. Thus, for an elementary reaction, the profile of *F*(*ξ*) exhibits a minimum at *ξ*_min_ and maximum at *ξ*_max_; we use here convention introduced by Politzer et al. [5] *ξ*_*min*_ ≡ *α*, *ξ*_*TS*_ ≡ *β*, *ξ*_*max*_ ≡ *γ*. These critical points define three reaction regions: the reactant (R; *ξ*_R_ ≤ *ξ* ≤ α), the transition state (TS; α ≤ *ξ* ≤ β) and the product (P; β ≤ *ξ* ≤ *ξ*_P_) regions [3, 5–7]. It has been shown in numerous examples [2–11] that most of the changes in the electronic structure usually take place in the TS region, while the reactant and the product regions are mostly characterized by structural changes (reactants preparation before TS, and relaxation of the structure after TS).

The decomposition of the reaction force into the strain and interaction components proposed recently by Politzer et al. [11] is based on the Activation Strain Model introduced by Bickelhaupt et al. [12–15]. In this model, the potential energy profile of the reaction, *E*(*ξ*) is decomposed into two contributions accounting for the structural deformation of fragments (A and B), Δ*E*_*strain*_(*ξ*) = Δ*E*_*A*_(*ξ*) + Δ*E*_*B*_(*ξ*), and the interaction between them, Δ*E*_*int*_(*ξ*):

2 $$ E\left(\xi \right)=\Delta {E}_{strain}\left(\xi \right)+\Delta {E}_{int}\left(\xi \right) $$

Differentiation of Eq. (2) with respect to *ξ* leads to [11]:

3 $$ F\left(\xi \right)=-\frac{\mathrm{d}E\left(\xi \right)}{d\xi}=-\frac{\mathrm{d}\Delta {E}_{strain}\left(\xi \right)}{d\xi}-\frac{\mathrm{d}\Delta {E}_{int}\left(\xi \right)}{d\xi}\equiv {F}_{strain}\left(\xi \right)+{F}_{int}\left(\xi \right) $$

It is worth emphasizing that the strain and the interaction forces, *F*_*strain*_(*ξ*) and *F*_*int*_(*ξ*), always work in opposite direction. In particular, at the TS structure, the strain and interaction forces compensate each other: *F*_*strain*_(*β*) = −*F*_*int*_(*β*), since *F*(*β*) = 0.

In the ETS-NOCV decomposition of the reaction force [16], further partitioning of the interaction term is considered, following the ETS bond-energy decomposition [17–19]:

4 $$ \Delta {E}_{int}\left(\xi \right)=\Delta {E}_{elstat}\left(\xi \right)+\Delta {E}_{Pauli}\left(\xi \right)+\Delta {E}_{orb}\left(\xi \right) $$

and the ETS-NOCV analysis [20]:

5 $$ {\varDelta E}_{int}\left(\xi \right)={\varDelta E}_{elstat}\left(\xi \right)+{\varDelta E}_{Pauli}\left(\xi \right)+\sum \limits_k^{M/2}\Delta  {E}_{orb,k}\left(\xi \right) $$

The first term in Eq. (4) corresponds to the electrostatic interaction between the two fragments in the supermolecule geometry, Δ*E*_*elstat*_(*ξ*). The Pauli repulsion term, Δ*E*_*Pauli*_(*ξ*), is the repulsive interaction between occupied orbitals of the two fragments, and the orbital interaction term. Finally, Δ*E*_*orb*_(*ξ*), represents the stabilizing component due to the final orbital relaxation. It is worth pointing out that the orbital interaction term includes both the inter- and intra-fragment polarizations: the former originates from the interaction of the occupied orbitals of one fragment with virtual orbitals of the other subsystem, while the latter emerges from mixing of the occupied and virtual orbitals of the same fragment.

In the ETS-NOCV analysis, the orbital interaction energy is further decomposed into the contributions *∆E*_*orb*, *k*_(*ξ*) (see Eq. 5), corresponding to pairs of *Natural Orbitals for Chemical Valence* (NOCV, ***ψ***) that provide the orbital representation of the deformation density, *∆ρ* = *ρ*_*AB*_(*r*) − [*ρ*_*A*_(*r*) + *ρ*_*B*_(*r*)]:

6 $$ \varDelta \rho (r)=\sum \limits_{k=1}{v}_k\left[-{\psi}_{-k}^2(r)+{\psi}_k^2(r)\right]=\sum \limits_{k=1}\varDelta {\rho}_k(r) $$

In Eq. (6) NOCV *ψ*_−*k*_ and *ψ*_*k*_ are the eigenvectors of the *∆* ***P*** matrix corresponding to eigenvalues ±*ν*_*k*_, where *∆* ***P*** = ***P***_*AB*_ − (***P***_*A*_ + ***P***_*B*_), and ***P***_*AB*_, ***P***_*A*_, and ***P***_*B*_ are the molecular, and the fragment *charge-and-bond-order* matrices, for details see Refs. [20, 24, 25].

Differentiation of Eqs. (4) and (5) with respect to the reaction coordinate leads to decomposition of the reaction force into the ETS / ETS-NOCV components:

7 $$ {F}_{int}\left(\xi \right)={F}_{elstat}\left(\xi \right)+{F}_{Pauli}\left(\xi \right)+{F}_{orb}\left(\xi \right)={F}_{elstat}\left(\xi \right)+{F}_{Pauli}\left(\xi \right)+\sum \limits_k{F}_{orb,k}\left(\xi \right) $$

where *F*_*elstat*_(*ξ*) = − *dΔE*_*elstat*_(*ξ*)/*dξ*, *F*_*Pauli*_(*ξ*) =  − *dΔE*_*Pauli*_(*ξ*)/*dξ*, *F*_*orb*_(*ξ*) =  − *dΔE*_*orb*_(*ξ*)/*dξ*, and *F*_*orb*, *k*_(*ξ*) =  − *dΔE*_*orb*, *k*_(*ξ*)/*dξ*.

The decomposition schemes described above assume partitioning of the system into the fragments. We have emphasized in previous papers [16, 21, 26] that fragments that arise as a natural choice for a description of the initial reactant region may not be so intuitive for the structures appearing in other regions of the reaction pathway, e.g., the products, or the transition state. What follows, the changes of the electronic structure, can be described based on different fragmentation schemes, e.g., ‘from the reactants perspective’ or ‘from the products perspective’. Also, depending on the partitioning of the system, the same effects may be included in different terms resulting from the energy/reaction force decomposition. One must be aware that this certainly introduces some arbitrariness. On the other hand, it is possible to choose the fragmentation scheme suitable for specific purpose, e.g., focusing on the bond formation or bond-breaking processes, and thus, providing the complementary picture of the changes in the electronic structure [16, 21].

### Computational details

All of the structures have been optimized in DFT/B3LYP calculations [27–29] with 6–31 + G(d,p) standard basis sets using the Gaussian 09 [30] program. The stationary points were verified by frequency calculations. Following the TS optimization, the intrinsic reaction coordinate (IRC) paths from TS towards the reactants and the products were determined. The ETS-NOCV analysis was performed for the IRC points based on the DFT/B3LYP calculations with standard triple-ζ basis with two sets of polarization functions (TZ2P) using the Amsterdam Density Functional (ADF) program [31–33].

## Results and discussion

**Fig. 2:**
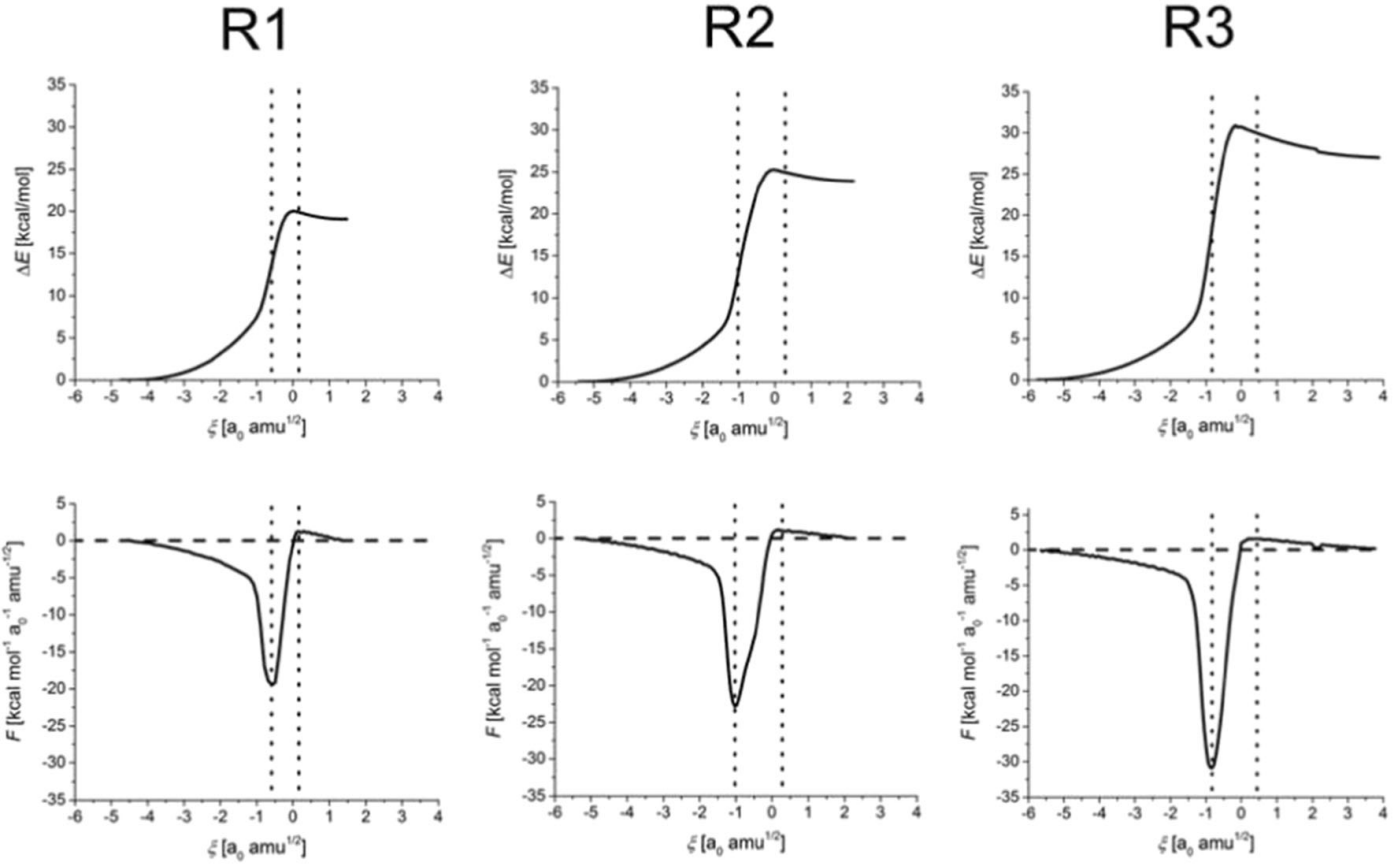
IRC energy and the reaction force profiles for R1, R2, R3 (see Fig. 1). *Vertical lines* indicate the borders between the reaction regions (reactant-, TS- and product region) defined by the extrema of the reaction force (at *ξ = α* and *ξ = γ*)

The IRC energy and the reaction force profiles for the three reactions studied (see Fig. 1) are displayed in Fig. 2; selected numerical characteristics of the reaction paths are collected in Supporting Information (Table S1). Comparing the three systems, the activation barrier increases in the order: formamide dimer < formamide–thioformamide system < thioformamide dimer. Accordingly, the minimum value of the reaction force is becoming more negative (retarding) in the same order. These results are in agreement with the earlier studies by Inostroza-Riviera et al. [23].

**Fig. 3:**
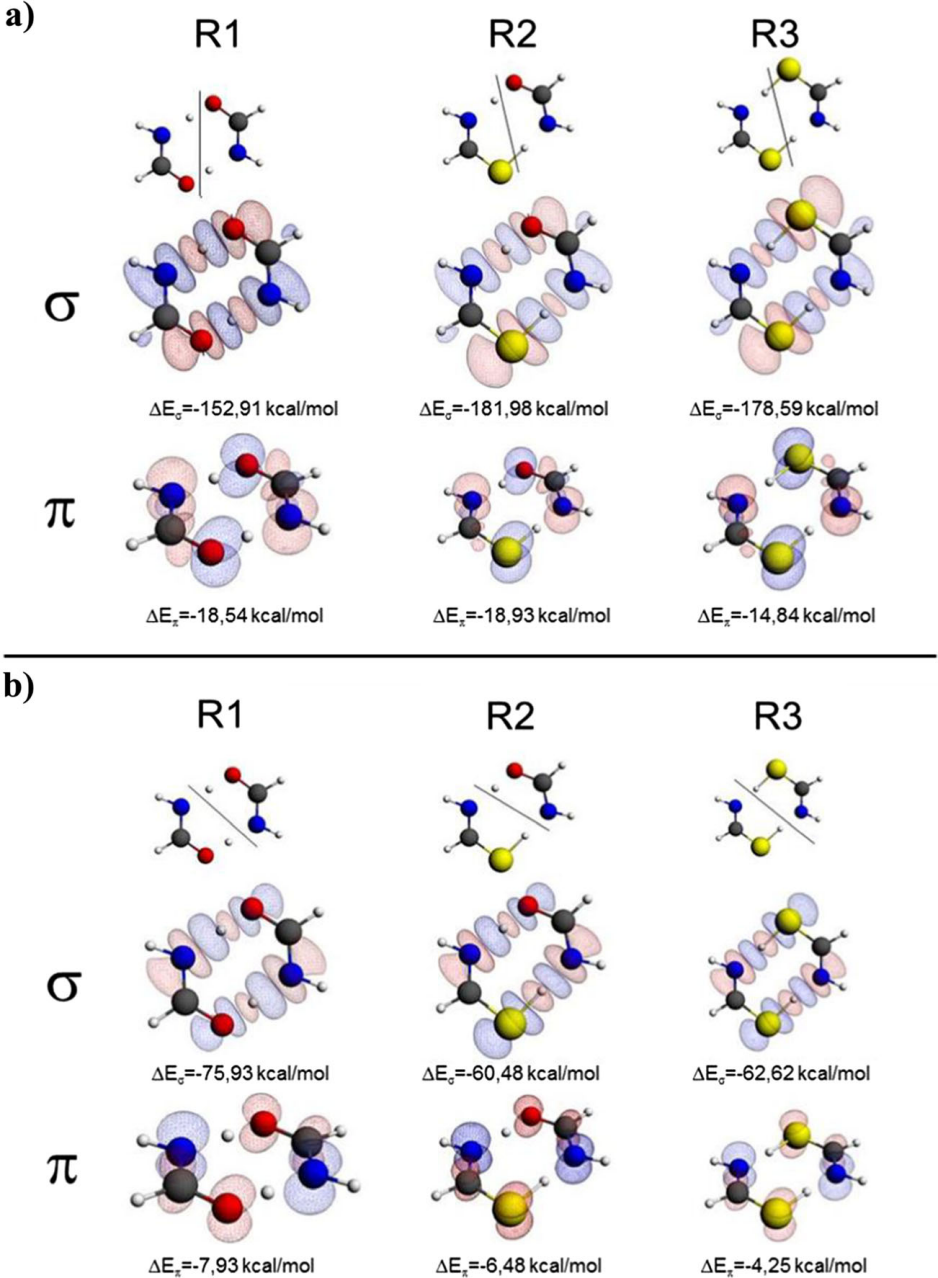
ETS-NOCV σ- and π- deformation-density contributions, Δ*ρ*_*σ*_ and Δ*ρ*_*π*_, for the TS structures using the ‘reactant’ (part **a**) and ‘product’ (part **b**) partitioning scheme (isovalue = 0.0005 a.u.). *Red contours* correspond to depletion (outflow) of electron density whereas *blue color* indicates the electron density accumulation (inflow). The ‘reactant’ and ‘product’ fragmentation schemes are indicated by the *solid line* dividing the structure into two fragments: in the ‘reactant perspective’ (part **a**) the NH_2_CHO or NH_2_CHS fragments are considered, while in the ‘product perspective’ (part **b**) the system is divided into the NHCHOH or NHCHSH fragments

Before discussing the results of decomposition of the reaction force, let us comment of the fragments considered for this purpose. Two fragmentation schemes applied here are displayed in Fig. 3, together with the results of the ETS-NOCV analysis. In the case of R1, the fragmentation of the supermolecule into two formamide molecules (or two thioformamide or formamide / thioformamide, for R2, R3, respectively) is natural for the initial stage of the reaction (‘reactant perspective’, see part **a** of Fig. 3), while choice of the two tautomeric forms (NH-CH-OH / NH-CH-SH) is natural for the final stage of reaction (‘product perspective’, see part **b** of Fig. 3). These two perspectives are considered in the present work, as they provide complementary picture of the changes in the electronic structure. Namely, in the case of the “reactant partitioning”, the interaction term is focused on the bond-formation processes: along the reaction paths the interaction between considered fragments leads to formation of the O-H bonds (as the O and H atoms forming the bonds are in two different fragments). The bond-breaking processes are scheme described by the strain component in this fragmentation (as both the N-H atoms are included in the same fragments). This is opposite in the “product partitioning”, where the interaction term is focused on the bond breaking (N-H atoms are in separate fragments), while bond formation is ‘hidden’ within the strain component (the O and H atoms forming the bond are in the same fragment).

In order to illustrate this point, in Fig. 3 we present results of the ETS-NOCV analysis performed for the TS geometries using the two partitioning schemes (‘reactant’ and ‘product’); the detailed intermediate NOCV results for formamide dimer are presented in Supporting Information (Figs. S1-S4). It is worth emphasizing that the NOCV deformation-density components calculated for the two partitioning schemes show ‘opposite’ patterns. Namely, for the ‘reactant’ partitioning, the σ components of Δ*ρ* indicates the presence of the OH (or SH) bonds (accumulation of the density between these two atoms, see Fig. 3a), while for the ‘product’ partitioning, the corresponding plots illustrate the presence of NH bonds (accumulation of the density between N and H atoms, see Fig. 3b). Accordingly, the accompanying π components of Δ*ρ* show accumulation of the density on the oxygen/sulfur atoms for ‘reactant’ partitioning (see Fig. 3a), and on the nitrogen atoms for the ‘product’ partitioning (see Fig. 3b).

**Fig. 4:**
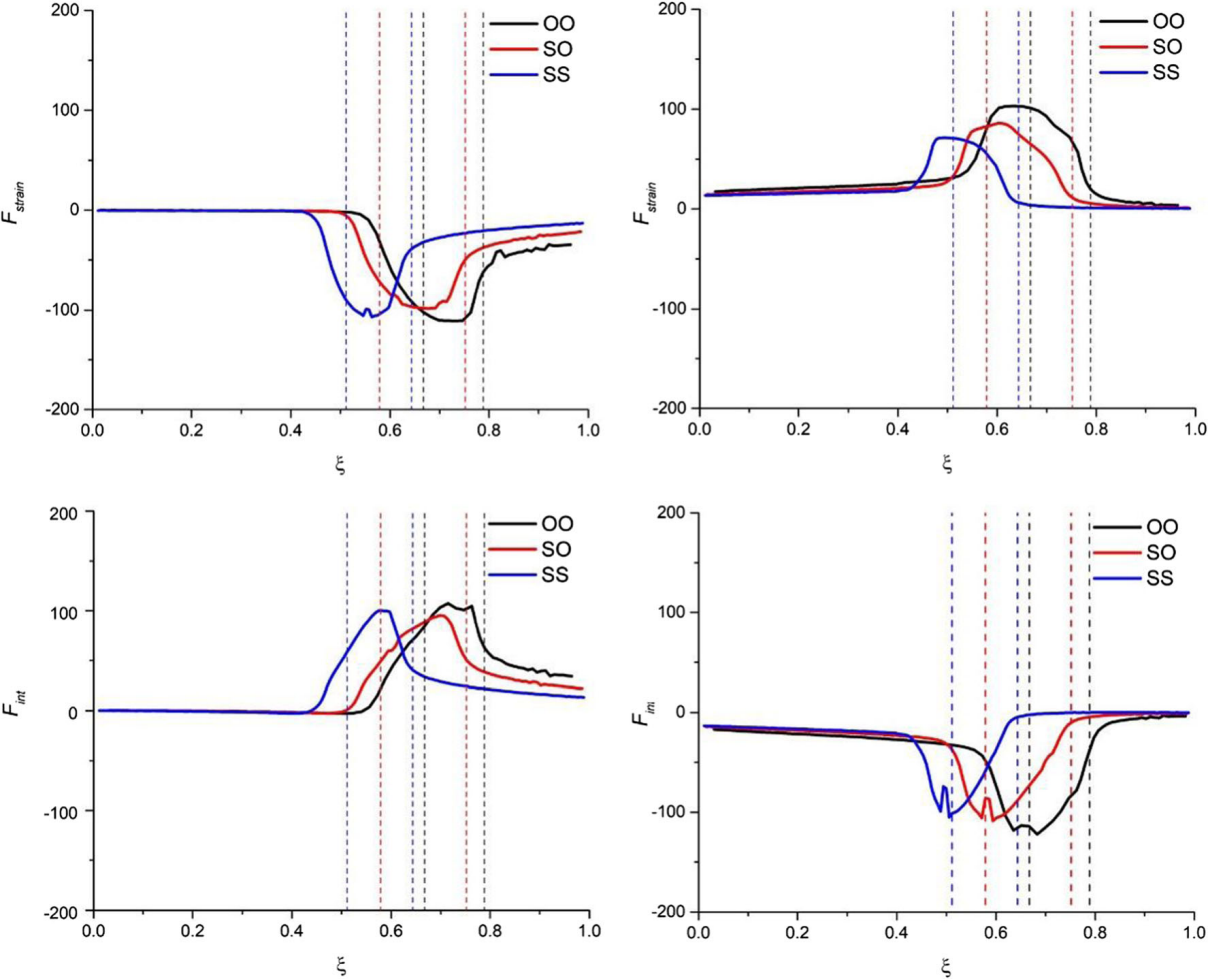
The reaction force components: strain force (top part, *F*_*strain*_(*ξ*)) and the interaction force (bottom part, *F*_*int*_(*ξ*)) for the ‘reactant’ partitioning (*left part*) and the ‘product’ partitioning (*right part*) along the reaction paths of double proton transfer in formamide dimer (OO), formamide–thioformamide system (OS) and thioformamide dimer (SS). Forces in [kcal ^.^ mol^−1 .^ a_0_^−1 .^ amu^−1/2^]

**Fig. 5:**
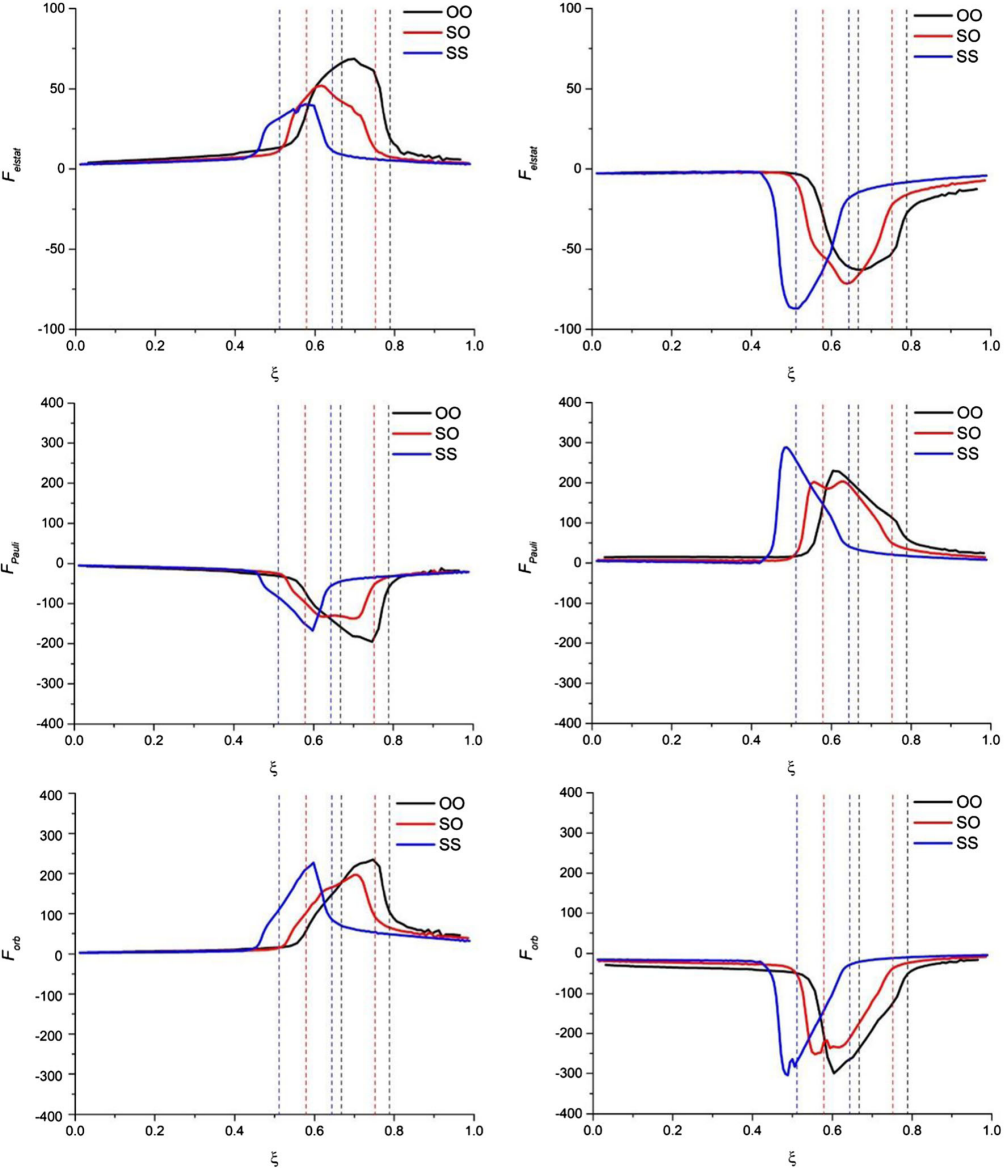
The reaction force components: electrostatic force (*top part*, *F*_*elstat*_(*ξ*)), Pauli-repulsion force (*middle part*, *F*_*Pauli*_(*ξ*) ) and the orbital-interaction force (*bottom part*, *F*_*orb*_(*ξ*)) for the ‘reactant’ partitioning (*left part*) and the ‘product’ partitioning (*right part*) along the reaction paths of double proton transfer in formamide dimer (OO), formamide–thioformamide system (OS) and thioformamide dimer (SS). Forces in [kcal ^.^ mol^−1 .^ a_0_^−1 .^ amu^−1/2^]

**Fig. 6:**
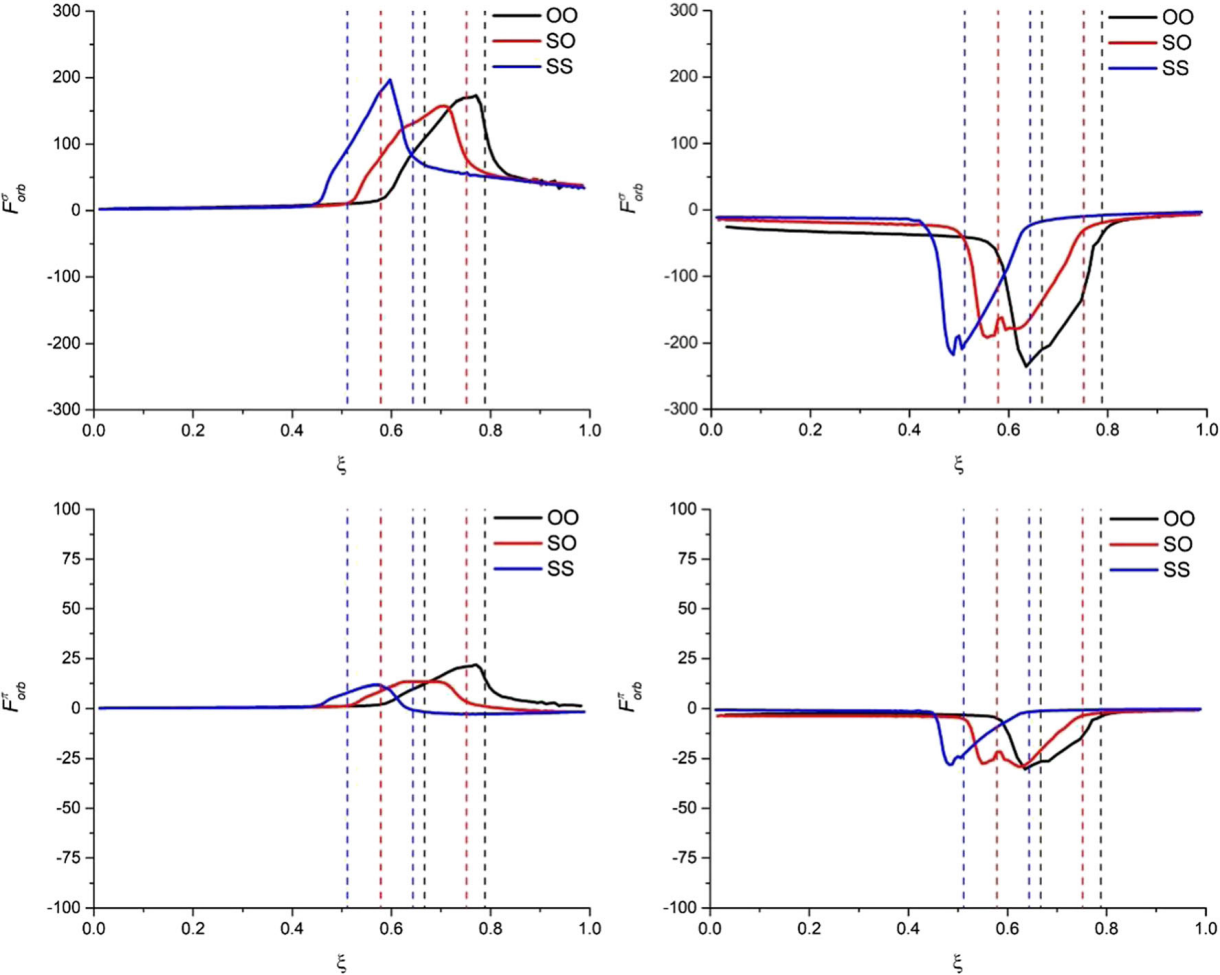
The σ- (*top part*) and π- (*bottom part*) contributions to *F*_*orb*_(*ξ*) for the ‘reactant’ partitioning (*left part*) and the ‘product’ partitioning (*right part*) along the reaction paths of double proton transfer in formamide dimer (OO), formamide–thioformamide system (OS) and thioformamide dimer (SS). Forces in [kcal ^.^ mol^−1 .^ a_0_^−1 .^ amu^−1/2^]

**Fig. 7:**
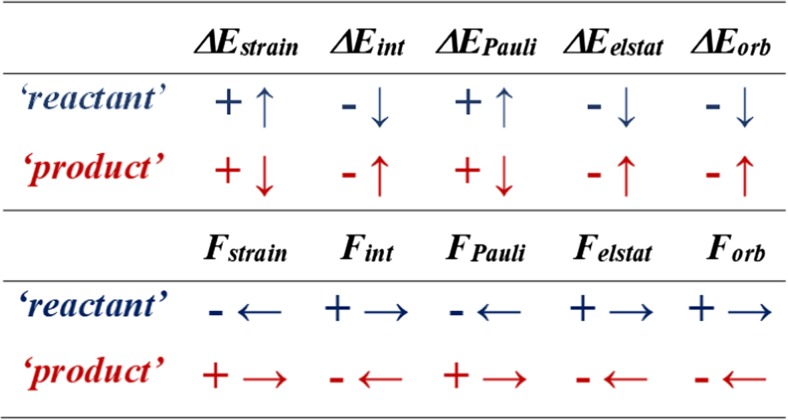
Schematic summary of the main features of the reaction energy and reaction force components for the ‘reactant’ and ‘product’ partitioning. For each component, the sign is presented (+/−); in the case of energy terms, the monotonicity is represented by ↑ and ↓ (increasing / decreasing along the pathway); in the case of the reaction force component, the driving/retarding character is indicated by → and ←

Results of decomposition of the reaction force for the ‘reactant’ and ‘product’ partitioning are collected in Fig. 4 (strain and interaction components), Fig. 5 (electrostatic, Pauli-repulsion, and orbital-interaction terms), and Fig. 6 (σ- and π- orbital-interaction contributions). The profiles of the corresponding energy terms are presented in Supporting Information Figs. S5, S6, and S7, respectively). Figure 7 schematically summarizes the character of respective components of energy and the reaction force for both partitioning schemes.

For the ‘reactant’ partitioning the strain energy, Δ*E*_*strain*_(*ξ*), is positive and increasing along the reaction paths, while the interaction energy, Δ*E*_*int*_(*ξ*), is negative (stabilizing) and decreasing from R to P, as it mainly describes the O-H bond formation. Thus, the strain force, *F*_*strain*_(*ξ*) is negative (retarding), and the interaction force, *F*_*int*_(*ξ*), is positive (driving the reaction). The Pauli component of the interaction energy, Δ*E*_*Pauli*_(*ξ*), is positive and increasing, giving rise to the negative (retarding) contribution to the reaction force, *F*_*Pauli*_(*ξ*). The electrostatic, Δ*E*_*elstat*_(*ξ*), and orbital-interaction, Δ*E*_*orb*_(*ξ*), components of the interaction energy are negative and decreasing, leading to positive, *F*_*elstat*_(*ξ*), and *F*_*orb*_(*ξ*), reaction-force contributions (driving the reaction).

For the ‘product’ partitioning (see right-hand side of Figs. 4, 5, 6), the sign (and character) of the corresponding reaction-force components is exactly opposite. The strain component, *F*_*strain*_(*ξ*), is now positive (driving), since the (positive) strain energy, Δ*E*_*strain*_(*ξ*), is decreasing from R to P. The interaction component, *F*_*int*_(*ξ*), is negative (retarding) for this partitioning, since the interaction energy, Δ*E*_*int*_(*ξ*), increases along the reaction path. This corresponds to the N-H bond-breaking: in the reactant structures, the large negative values of Δ*E*_*int*_(*ξ*) are due to the N-H covalent bonds, being broken along the pathway; in the product structure the less-negative values of Δ*E*_*int*_(*ξ*) reflect mainly the hydrogen bonding (N·····H-O) between the NH-CH-OH (or NH-CH-SH) species. Accordingly, for the ‘product’ partitioning, the electrostatic and orbital-interaction contributions to the reaction force, *F*_*elstat*_(*ξ*), and *F*_*orb*_(*ξ*), are negative (retarding), while the Pauli-repulsion term, *F*_*Pauli*_(*ξ*), is positive (reaction driving).

It is worth emphasizing as well that all the components of the reaction force exhibit the largest changes inside or in the close vicinity of the transition-state region; this is true for both partitioning schemes. However, while for the ‘reactant’ partitioning the extrema of the interaction force, *F*_*int*_(*ξ*), and its components (*F*_*elstat*_(*ξ*), *F*_*Pauli*_(*ξ*), *F*_*orb*_(*ξ*)) are located inside the TS region, for the ‘product’ partitioning, they are located at the border of the TS region (*ξ* = α). This indicates that the bond-breaking processes start prior to the TS region, giving rise to the extremum of the retarding force.

**Fig. 8:**
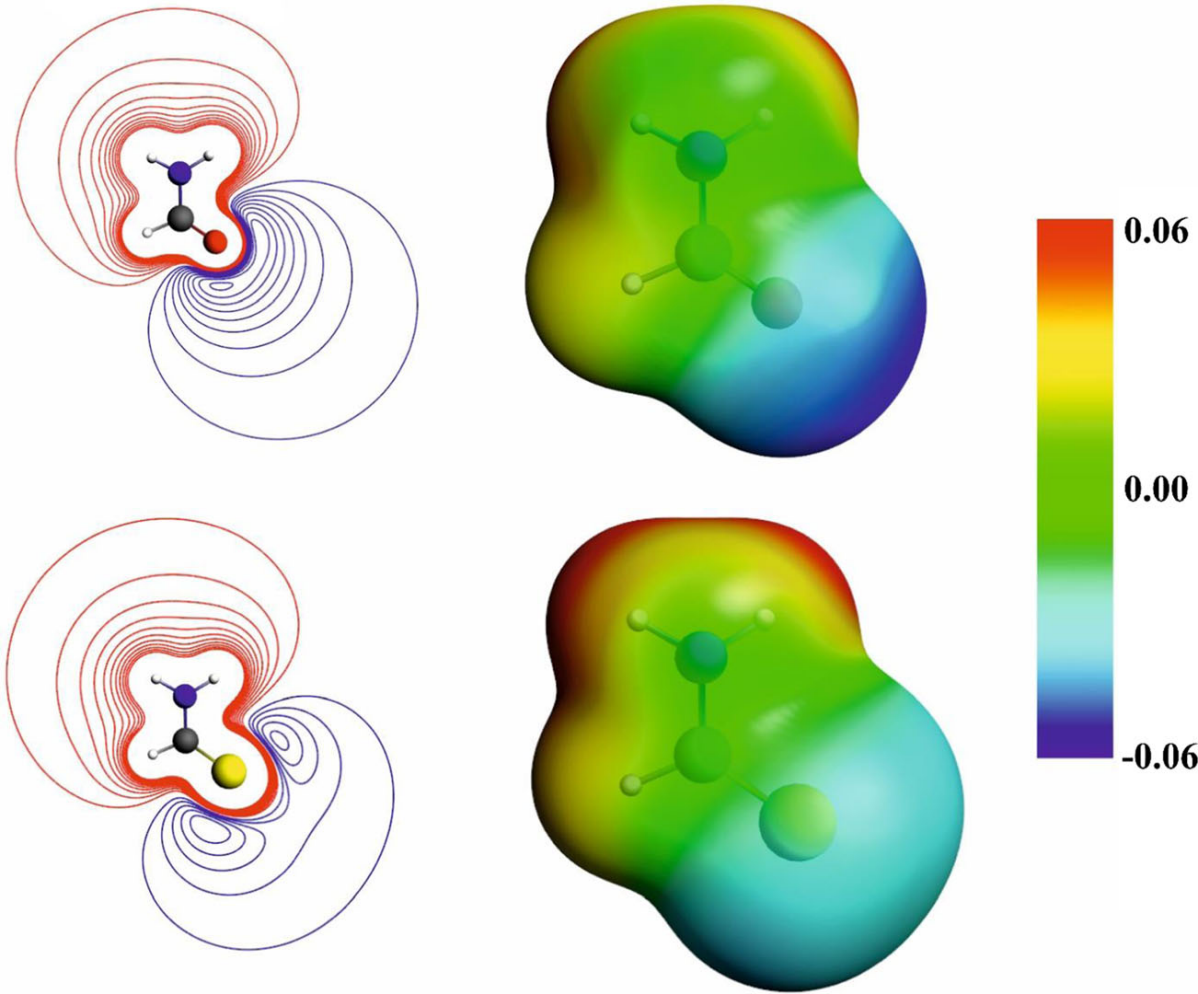
Comparison of the molecular electrostatic potential for formamide (*top*) and thioformamide (*bottom*): the contour maps (contour interval 0.01 a.u.; *blue lines* indicate negative, and *red* - positive values) and the color-coded representation on the electron density isosurface (ρ = 0.001; the color scale is shown on the right, in a.u.)

Finally, let us focus on the comparison of the reaction force components for the three considered reactions in the context of the increasing activation barrier: R1 < R2 < R3. From the strain and interaction components of the reaction force (see Fig. 4), it is hard to draw any conclusions about the main factors responsible for the barrier increase from R1 to R3. When going deeper into the interaction components of the reaction force (see Fig. 5), one can see that for both partitioning the *F*_*Pauli*_(*ξ*) and *F*_*orb*_(*ξ*) exhibit minima (maxima) in different order: lower (higher) for R1 and R2, and higher (lower) for R2. However, the electrostatic driving force accompanying the O-H / S-H bond-formation (*F*_*elstat*_(*ξ*) for ‘reactant’ partitioning) is decreasing in the order R1 > R2 > R3. This is quite intuitive, and can be explained by the difference in the molecular electrostatic potential of formamide and thioformamide: the transferred protons are more strongly stabilized by oxygen atoms than by sulfur atoms (Fig. 8). Moreover, the electrostatic retarding force accompanying N-H bond-breaking (*F*_*elstat*_(*ξ*) for ‘product’ partitioning) is as well growing (i.e., becoming more negative) in the order R1, R2, R3. Thus, the electrostatic component, associated with both bond formation and bond-breaking processes, appears to be the main factor responsible for the barrier increase from R1 to R3.

## Conclusions

In the present work, the analysis of the changes in the electronic structure along IRC paths for double-proton-transfer reactions in the formamide dimer (R1), formamide–thioformamide system (R2), and the thioformamide dimer (R3) was performed based on the ETS-NOCV partitioning of the reaction force. In this analysis, the intra-fragments strain and the inter-fragments interaction terms of ASM model are initially considered; the interaction term is further decomposed into the electrostatic, Pauli-repulsion, and orbital interaction components, with the latter being divided into the NOCV σ- and π- components. The ‘reactant perspective’ and ‘the product perspective’ have been considered, corresponding to two complementary ways of partitioning of the system into the molecular fragments: the former is ‘natural’ at the reactant stage, while the latter is ‘natural’ for the products; the two fragmentation schemes allow for discussion the bond-formation and bond-breaking processes. It should be emphasized that depending on the fragmentation of the system, the driving and retarding components are included in different ETS-NOCV reaction-force terms: in the ‘reactant’ partitioning, the electrostatic and orbital interactions contributions are positive (driving the reaction), and the strain and the Pauli-repulsion contributions are negative (acting against the reaction progress), while for the ‘product’ partitioning this is opposite.

The results indicate that the major changes in the electronic structure occur in the transition state region, in agreement with previous studies [22, 23]. The reaction force components for the ‘product’ partitioning, however, indicate that the bond-breaking processes are initiated already in the reactant region, prior to entering the TS region. Finally, the results show that the electrostatic contributions are the main factor responsible for the increase in the activation barrier in the order R1 < R2 < R3.

## Electronic supplementary material

ESM 1 (PDF 1338 kb)

## References

[1] Toro-Labbé A (1999). Characterization of chemical reactions from the profiles of energy, chemical potential, and hardness. J Phys Chem A.

[2] Jaque P, Toro-Labbé A, Politzer P, Geerlings P (2008). Reaction force constant and projected force constants of vibrational modes along the path of an intramolecular proton transfer reaction. Chem Phys Lett.

[3] Politzer P, Toro-Labbé A, Gutiérrez-Oliva S., Murray JS (2012) Perspectives on the reaction force. Adv Quantum Chem 64:189–209

[4] Politzer P, Murray JS, Jaque P (2013). Perspectives on the reaction force constant. J Mol Model..

[5] Politzer P, Toro-Labbé A, Gutiérrez-Oliva S (2005). The reaction force: three key points along an intrinsic reaction coordinate. J Chem Sci..

[6] Toro-Labbé A, Gutiérrez-Oliva S, Murray JS, Politzer P (2007). A new perspective on chemical and physical processes: the reaction force. Mol Phys..

[7] Toro-Labbé A, Gutiérrez-Oliva S, Murray JS, Politzer P (2009). The reaction force and the transition region of a reaction. J Mol Model..

[8] Toro-Labbé A, Gutiérrez-Oliva S, Concha MC (2004). Analysis of two intramolecular proton transfer processes in terms of the reaction force. J Chem Phys..

[9] Burda JV, Toro-Labbé A, Gutiérrez-Oliva S (2007). Reaction force decomposition of activation barriers to elucidate solvent effects. J Phys Chem A.

[10] Yepes D, Murray JS, Politzer P, Jaque P (2012). The reaction force constant: an indicator of the synchronicity in double proton transfer reactions. Phys. Chem. Chem Phys..

[11] Politzer P, Murray JS, Yepes D, Jaque P (2014). Driving and retarding forces in a chemical reaction. J Mol Model..

[12] Bickelhaupt FM (1999). Understanding reactivity with Kohn–Sham molecular orbital theory: E2-SN2 mechanistic spectrum and other concepts. J Comput Chem..

[13] Fernandez I, Bickelhaupt, FM (2014) The activation strain model and molecular orbital theory: understanding and designing chemical reactions. Chem Soc Rev 43:4953–4967 10.1039/c4cs00055b 24699791

[14] de Jong GT, Bickelhaupt FM (2007). Transition-state energy and position along the reaction coordinate in an extended activation strain model. Chem Phys Chem.

[15] van Bochove MA, Swart M, Bickelhaupt FM (2006). J Am Chem Soc..

[16] Díaz S, Brela MZ, Gutiérrez-Oliva S (2017). ETS-NOCV decomposition of the reaction force: the HCN/CNH isomerization reaction assisted by water. J Comput Chem..

[17] Ziegler T, Rauk A (1977). On the calculation of bonding energies by the Hartree–Fock–Slater method. Theor Chim Acta.

[18] Ziegler T, Rauk A (1979). Carbon monoxide, carbon monosulfide, molecular nitrogen, phosphorus trifluoride, and methyl isocyanide as .Sigma. Donors and .Pi. Acceptors. A theoretical study by the Hartree–Fock–Slater transition-state method. Inorg Chem..

[19] Ziegler T, Rauk A (1979). A theoretical study of the ethylene-metal bond in complexes between copper(1+), silver(1+), gold(1+), platinum(0) or platinum(2+) and ethylene, based on the Hartree-Fock–Slater transition-state method. Inorg Chem..

[20] Mitoraj MP, Michalak A, Ziegler T (2009). A combined charge and energy decomposition scheme for bond analysis. J Chem Theory Comput..

[21] Šebesta F, Brela MZ, Diaz S (2017). The influence of the metal cations and microhydration on the reaction trajectory of the N3 ↔ O2 thymine proton transfer: quantum mechanical study. J Comput Chem..

[22] Hargis JC, Vöhringer-Martinez E, Woodcock HL (2011). Characterizing the mechanism of the double proton transfer in the formamide dimer. J Phys Chem A.

[23] Inostroza-Rivera R, Herrera B, Toro-Labbé A (2014). Using the reaction force and the reaction electronic flux on the proton transfer of formamide-derived systems. Phys Chem Chem Phys..

[24] Mitoraj M, Michalak A (2007). Natural orbitals for chemical valence as descriptors of chemical bonding in transition metal complexes. J. Mol. Model..

[25] Michalak A, Mitoraj M, Ziegler T (2008). Bond orbitals from chemical valence theory. J Phys Chem A.

[26] Mitoraj MP, Parafiniuk M, Srebro M (2011). Applications of the ETS-NOCV method in descriptions of chemical reactions. J Mol Model..

[27] Becke AD (1993). Density-functional thermochemistry. III. The role of exact exchange. J Chem Phys.

[28] Lee C, Yang W, Parr RG (1988). Development of the Colle–Salvetti correlation-energy formula into a functional of the electron density. Phys Rev B.

[29] Stephens PJ, Devlin FJ, Chabalowski CF, Frisch MJ (1994). Ab initio calculation of vibrational absorption and circular dichroism spectra using density functional force fields. J Phys Chem..

[30] Frisch MJ, Trucks GW, Schlegel HB et al (2009) Gaussian 09 (revision a.01). Gaussian Inc, Wallingford

[31] te Velde G, Bickelhaupt FM, Baerends EJ (2001). Chemistry with ADF. J. Comput. Chem..

[32] Fonseca Guerra C, Snijders JG, te Velde G, Baerends EJ (1998). Towards an order- N DFT method. Theor Chem accounts theory. Comput Model (Theoretica Chim Acta).

[33] Baerends EJ, Ziegler T, Atkins AJ et al (2014) ADF2014, SCM, Theoretical Chemistry, Vrije Universiteit, Amsterdam. http://www.scm.com

