# Supplementary material for: ETS-NOCV decomposition of the reaction force for double-proton transfer in formamide-derived systems
Source: J Mol Model. 2017 Dec 22;24(1):27. doi: 10.1007/s00894-017-3564-9 (PMC5741796; doi:10.1007/s00894-017-3564-9)
Supplement: Supplementary file 1 — (PDF 1338 kb) [file 894_2017_3564_MOESM1_ESM.pdf]

Electronic supplementary material

ETS-NOCV Decomposition of the Reaction Force for the Double Proton-Transfer in Formamide-Derived Systems

Piotr Talaga, Mateusz Z. Brela, and Artur Michalak*

Department of Theoretical Chemistry, Faculty of Chemistry, Jagiellonian University,

Gronostajowa 2, 30-387 Krakow, Poland,

*E-mail: michalak@chemia.uj.edu.pl

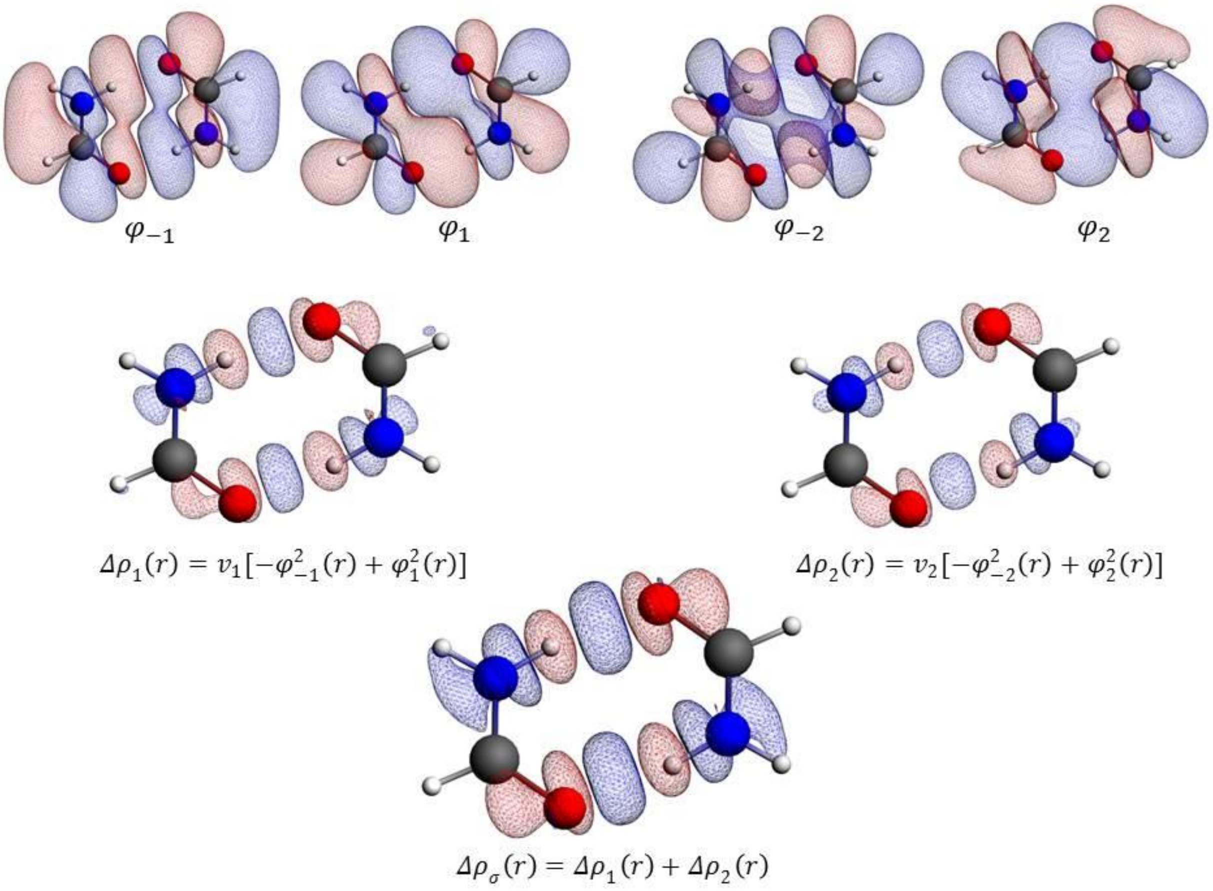

Figure S1. Natural orbitals for chemical valence (NOCV) and the resulting deformation density contributions (σ) for the 'reactant' partitioning.

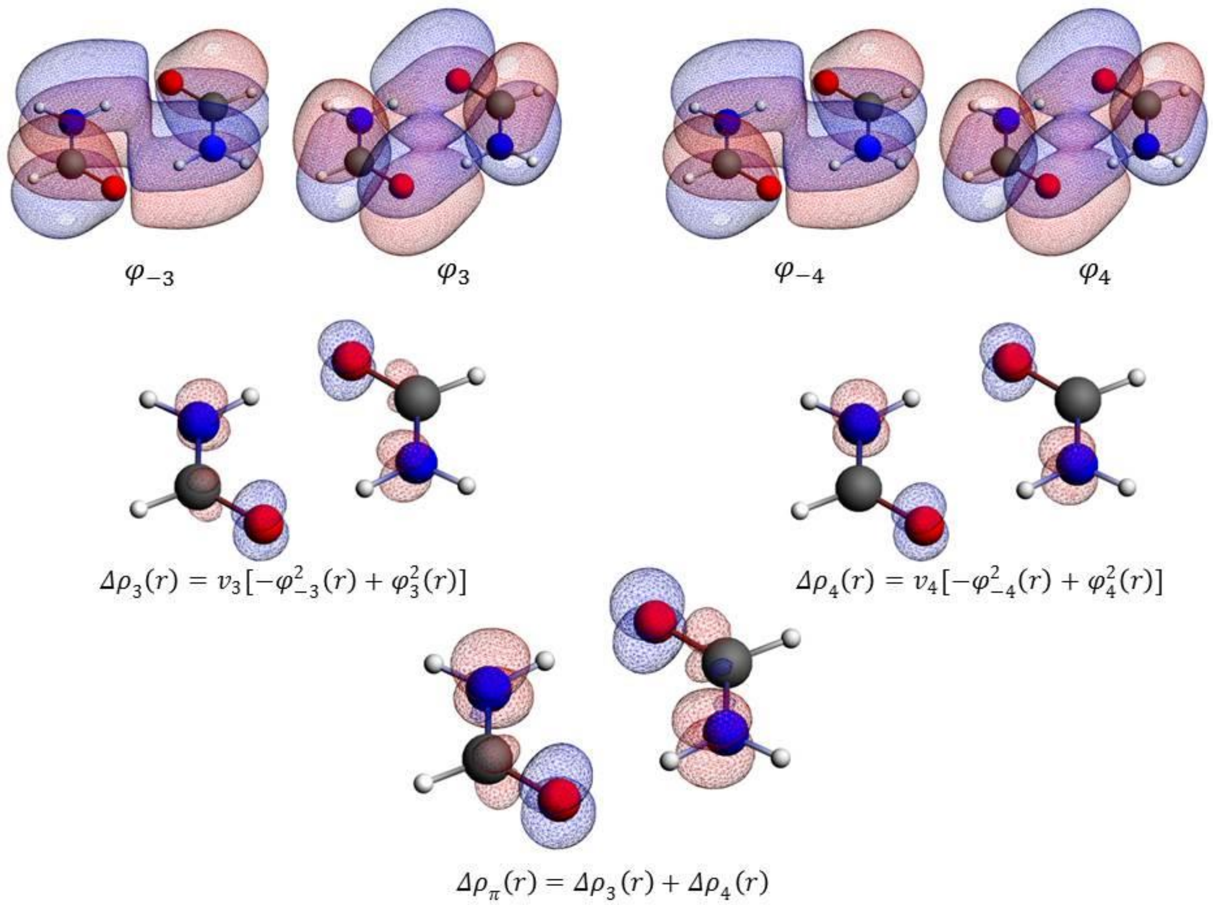

Figure S2. Natural orbitals for chemical valence (NOCV) and the resulting deformation density contributions (π) for the 'reactant' partitioning.

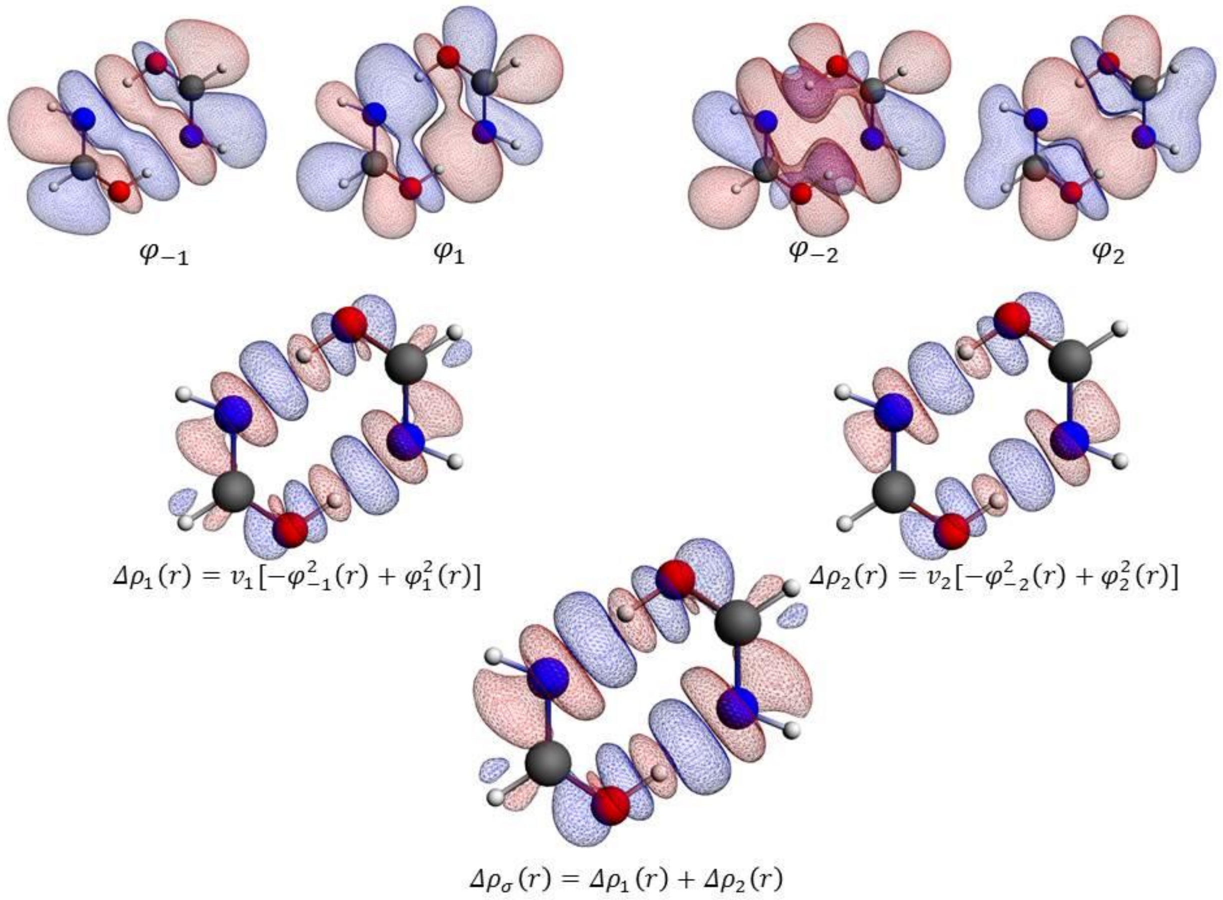

Figure S3. Natural orbitals for chemical valence (NOCV) and the resulting deformation density contributions (σ) for the 'product' partitioning.

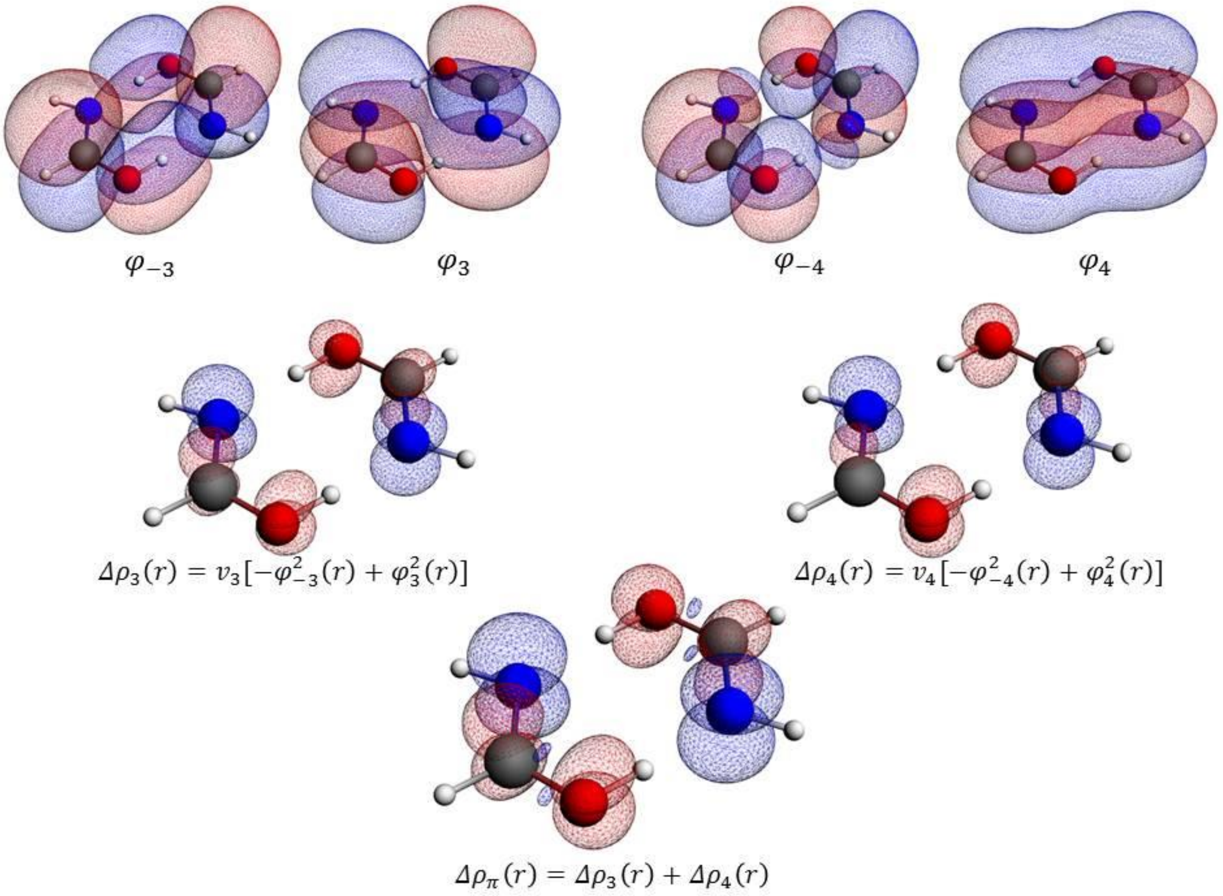

Figure S4. Natural orbitals for chemical valence (NOCV) and the resulting deformation density contributions (π) for the 'product' partitioning.

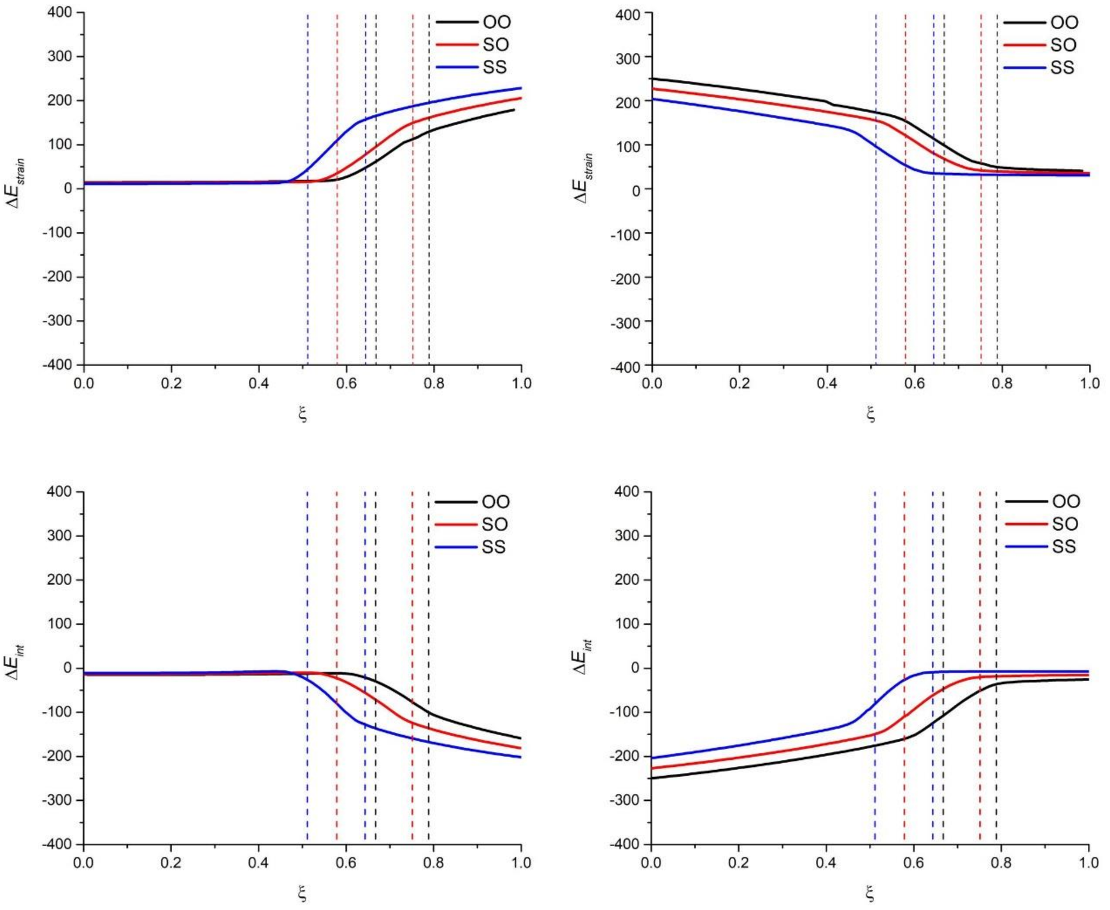

Figure S5. The energy components: strain (top part, $\Delta E_{strain}(\xi)$) and the interaction (bottom part, $\Delta E_{int}(\xi)$) for the 'reactant' partitioning (left part) and the 'product' partitioning (right part) along the reaction paths of double proton transfer in formamide dimer (OO), formamide / thioformamide system (OS) and thioformamide dimer (SS). Energy in kcal/mol.

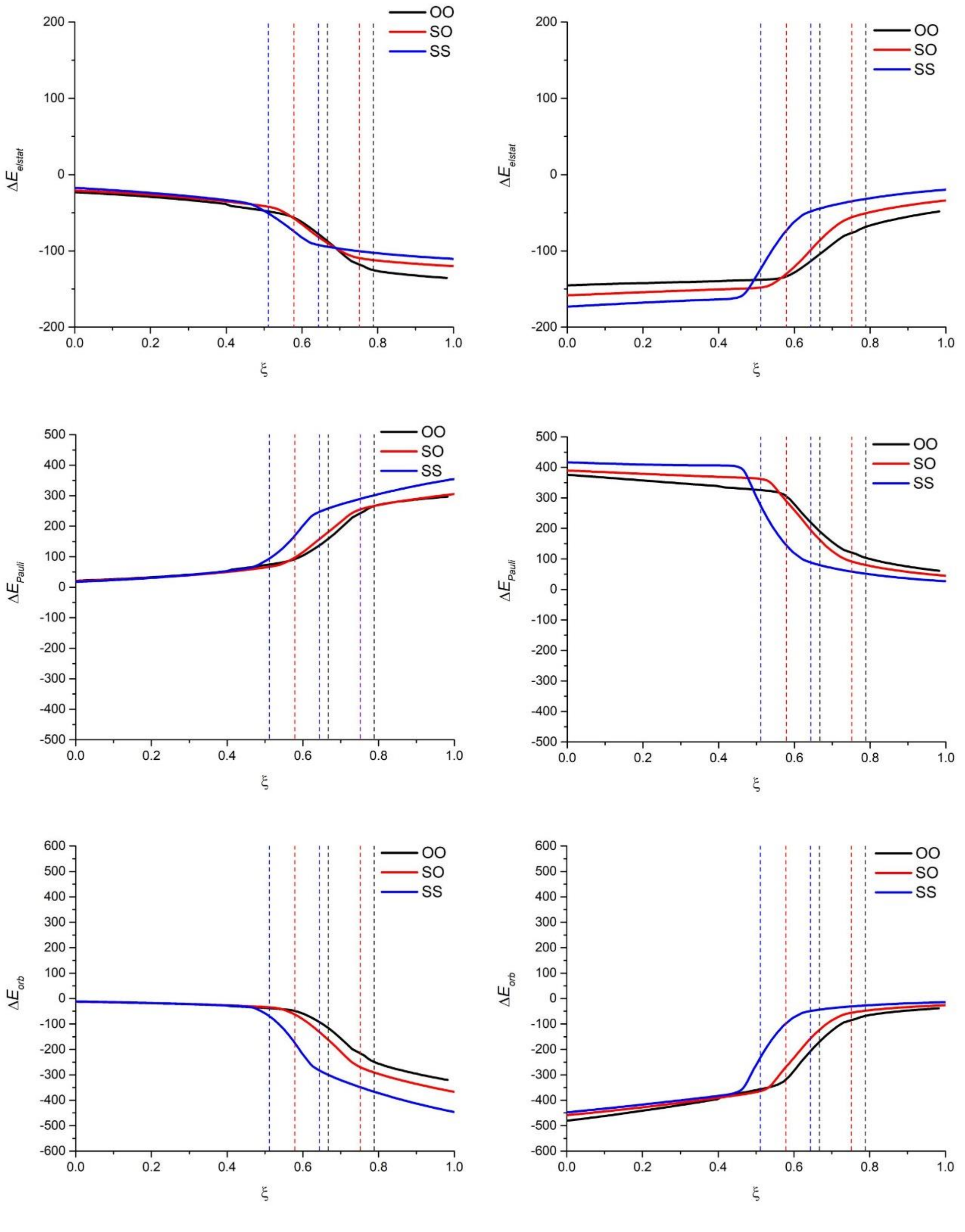

Figure S6. The energy components: electrostatic (top part, $\Delta E_{elstat}(\xi)$), Pauli-repulsion (middle part, $\Delta E_{Pauli}(\xi)$) and the orbital-interaction (bottom part, $\Delta E_{orb}(\xi)$) for the 'reactant' partitioning (left part) and the 'product' partitioning (right part) along the reaction paths of double proton transfer in formamide dimer (OO), formamide / thioformamide system (OS) and thioformamide dimer (SS). Energy in kcal/mol.

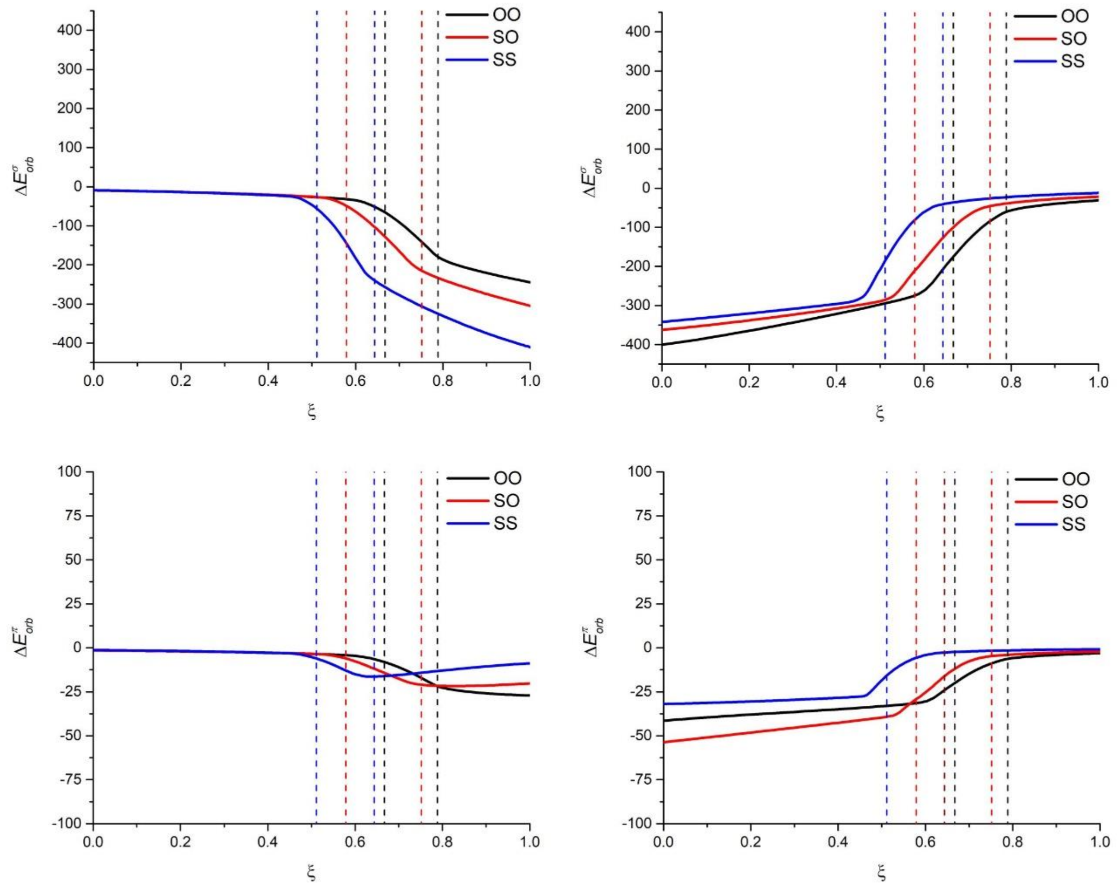

Figure S7. The σ- (top part) and π- (bottom part) contributions to $\Delta E^{\sigma}_{orb}(\xi)$ for the 'reactant' partitioning (left part) and the 'product' partitioning (right part) along the reaction paths of double proton transfer in formamide dimer (OO), formamide / thioformamide system (OS) and thioformamide dimer (SS). Energy in kcal/mol.

Table S1. Selected characteristics of the IRC and reaction force profiles for reactions **R1, R2, R3**

	R1	R2	R3
Activation barier [a]	20.01	25.23	30.72
Reaction energy [a]	19.08	23.87	27.01
Reaction coordinate for reactant (R) [b]	-4.74 (0.00)	-5.42 (0.00)	-5.75 (0.00)
Reaction coordinate for F_{max} (α) [b]	-0.59 (0.67)	-1.03 (0.58)	-0.83 (0.51)
Reaction coordinate for TS (β) [b]	0.00 (0.76)	0.00 (0.71)	0.00 (0.60)
Reaction coordinate for F_{min} (γ) [b]	0.16 (0.79)	0.29 (0.75)	0.44 (0.64)
Reaction coordinate for the product (P) [b]	1.48 (1.00)	2.17 (1.00)	3.87 (1.00)
F_{max} value [c]	-19.49	-22.86	-30.91
F_{min} value [c]	1.22	1.07	1.54

[a] in [kcal/mol]

[b] in [a_0 $amu^{1/2}$]; (in parenthesis: dimensionless, with respect to the reactant)

[c] in [kcal mol^{-1} a_0^{-1} $amu^{-1/2}$]
